# Factors influencing drug switching and changes in low-density lipoprotein-cholesterol levels with atorvastatin: a real-world observational study

**DOI:** 10.1186/s12944-023-01903-2

**Published:** 2023-09-13

**Authors:** Yu-Cheol Lim, Eui-Kyung Lee, Mi-Hai Park

**Affiliations:** School of Pharmacy, Sungkyunkwan University, Suwon, Gyeonggi-Do South Korea

**Keywords:** Drug switching, Generic drug, Atorvastatin, Low-density lipoprotein-cholesterol, Real-world data

## Abstract

**Background:**

Although generic drugs have been approved with the assurance of interchangeable applications with original drugs, some physicians, and patients still view their efficacy and interchangeability negatively. Using real-world data, we aimed to determine factors that impact switching between drugs that contain the same active ingredient, i.e., atorvastatin, and, in turn, whether this ‘switch’ could alter clinical outcomes.

**Methods:**

Using the National Health Insurance Service senior cohort, a retrospective cohort study was conducted to assess patients who had newly started atorvastatin 10 mg and had at least two records of national health examinations from 2010 to 2014. Drug switching, which was defined as a change in the atorvastatin product administered 90 days before the first and second examinations, was assessed. Greedy propensity score matching (1:2) was performed between switchers and non-switchers to control for potential confounders. Factors influencing switching were analyzed using multivariate logistic regression to estimate odds ratios and 95% confidence intervals (CIs). Changes in low-density lipoprotein-cholesterol (LDL-C) levels attributable to drug switching were evaluated using difference-in-differences regression.

**Results:**

A total of 1,588 patients were included, of whom 25.3% switched drugs (1,187 non-switchers and 401 switchers). Compared to patients taking generics before the first examination, those taking the original drugs had a lower odds ratio (0.31; 95% CI [0.21, 0.46]) for subsequent drug switching. A change in medical institution was associated with a significantly higher odds ratio (6.83; 95% CI [4.66, 10.02]). There were no significant differences in LDL-C alterations between switchers and non-switchers (0.42 mg/dL; 95% CI [-2.29, 3.13]).

**Conclusion:**

The type of first-time drug administered and changes in medical institution can influence drug switching. No significant changes in LDL-C values were observed in the various switching scenarios between the original and generic drugs, suggesting their interchangeable application in real-world clinical practice.

**Supplementary Information:**

The online version contains supplementary material available at 10.1186/s12944-023-01903-2.

## Background

A generic drug is one whose active ingredients are equivalent to those of the original drug (also known as the reference or brand-name drug), has passed bioequivalence testing, and has been approved by regulatory authorities for interchangeable applications with the original drug [1–3]. Generics typically enter the market at a lower price than that of the original drugs; therefore, they provide numerous benefits such as reduced healthcare costs and enhanced patient access to medical care. Because of these benefits, most developed countries have implemented policies to encourage generic drug prescriptions, including sanctions for budget overruns and incentives [4]. Accordingly, the generic market has steadily expanded, and numerous generics, along with original drugs, are prescribed in clinical settings to treat patients. For example, in the Netherlands, generic drugs account for 75.6% of the prescribed market share using International Nonproprietary Names [5].

However, because generic drugs can be approved by confirming their bioequivalence without undergoing clinical trials, whether their clinical effects are equivalent to those of the original drug when administered in clinical settings remains controversial [6, 7]. In a systematic review and meta-analysis comparing the safety and efficacy of original and generic cardiovascular drugs in 30 randomized controlled trials and 42 non-randomized clinical trials or observational studies, 60% of the studies identified no difference in effectiveness between original and generic administration, 26% found the original to be more efficacious and safer than the generic version, and 1% found the generic to be more effective than the original drug [8]. Conversely, a retrospective analysis utilizing real-world data found no significant clinical differences between patients prescribed five statin generics and the original drugs [9]. Additionally, Medicare patients treated with generic statins showed high drug compliance and low cardiovascular risk [10].

Previous studies have assessed the clinical effects of original and generic drugs in parallel groups, comparing original versus generic drugs, but not interchangeability. However, in real-world clinical settings, patients may be prescribed generics from several manufacturers, including the original drug, and switching within those drugs (e.g., original → generic, generic → another generic) may occur owing to various factors, including policy, social and economic factors, or clinician and patient choice [11, 12]. To date, no study has explored the differences in clinical effects associated with various real-world drug switch scenarios, including switching between generic drugs. Therefore, we attempted to determine the interchangeability of drugs administered over a prolonged period with the same active ingredients, such as atorvastatin, a commonly prescribed generic drug. This study investigated the factors influencing drug switching in older patients who began atorvastatin treatment. Furthermore, changes in low-density lipoprotein-cholesterol (LDL-C) levels were compared between switchers and non-switchers and in different drug-switching scenarios.

## Methods

### Data source

This study used National Health Insurance Service (NHIS)-senior cohort data between January 1, 2002, and December 31, 2015. The data source contained information on 558,147 patients, comprising 10% of the total population of approximately 5.5 million people aged ≥ 60 years. The recorded information included sex, age, medical services (inpatient and outpatient care), diagnostic codes, drug prescriptions, and healthcare providers [13]. Considering drug-related information, the product and active ingredient codes for each drug ingredient and dose were included. Furthermore, health examination records were linked to the national health screening program, which included test results such as physical checkups and blood tests including LDL assessment and chest X-rays. Because beneficiaries are recommended examinations once every two years, it was possible to analyze multiple examination records per patient during the observation period [14]. The related materials and metadata are publicly available on the National Health Insurance Data Sharing Service homepage (http://nhiss.nhis.or.kr).

### Study design and population

Herein, claims data for the study cohort from January 1, 2008, to December 31, 2015, were analyzed. The cohort entry period was defined as January 1, 2010, to December 31, 2014, to select patients who were new users of statins and ensure an examination gap of at least one year (Supplementary Fig. 1). Patients were included if they had been prescribed at least 10 mg atorvastatin during the cohort entry period and had not taken any lipid-lowering drugs during the prior two years. The inclusion criteria were as follows: patients who had at least two LDL-C tests recorded at the end of the observation period and those who had taken atorvastatin 90 days before the first (index date1) and second examinations (index date2). The 90-day statin-taking period was conservatively defined as the minimum exposure period to measure LDL-C changes, considering 4–6 weeks to be the period during which statins reach their maximum effect [15, 16]. Exclusion criteria were as follows: (1) patients who had changed to medications other than atorvastatin 10 mg during the 90-day period prior to each examination, (2) patients who had an atorvastatin medication compliance rate (MPR) of <0.8 in the 90-day period prior to each examination, and (3) patients whose LDL-C levels were outliers at either of the two examinations.

### Study outcomes and variables

Switchers were defined as patients who changed the atorvastatin product 90 days before index date1 or date2. Non-switchers were defined as patients who were prescribed the same product during both periods. There were three scenarios for switchers: from the original to generics, from generics to original drugs, and from generics to other generics; for non-switchers, there were two scenarios: prescribed original and generic drugs during the 90-day period before each index date.

The factors influencing drug-switching status were evaluated as dependent variables. The independent variables were selected based on previous drug switching and prescription behavior studies. The independent variables included demographic (age, sex), socioeconomic (income, medical institution), clinical (obesity, Charlson comorbidity index [CCI] score, co-medication, comorbidity) [17, 18], and behavioral factors (type of first-time drug prescribed, type of prescribing medical institution) [19, 20].

The mean LDL-C level at each index date and the mean change in LDL-C levels between the first and second examinations were calculated for each switch scenario group. The effect of drug switching on changes in LDL-C levels was assessed using the difference-in-differences (DID) method. In the DID analysis, baseline patient characteristics on the first examination date (index date1) and the CCI score from medical utilization records for the year before the first examination date were included as time-independent variables. Concomitant medications that may affect LDL-C levels were measured as cardiovascular, endocrine, and hormonal medications during the 90-day period prior to index date1 and date2 [21]. Comorbidities that may affect LDL-C levels were evaluated as ischemic heart disease, stroke, arteriosclerosis, diabetes mellitus, and congestive heart failure in the year prior to both index dates [22]. To compare the mean LDL-C change, a subgroup analysis was performed by dividing the patient population according to the factors influencing drug switching.

### Statistical analysis

To control for potential confounders between switchers and non-switchers, 1:2 greedy propensity score matching was performed for sex, age, household income quintile, CCI, and interval between LDL tests. Differences in characteristics between the switcher and non-switcher groups before and after propensity score matching are presented as standardized mean differences. Categorical variables, including sex, age group, household income quintile, CCI, obesity status, concomitant medications, and comorbidities, are presented as numbers of patients and proportions, while continuous variables, including age, days between LDL-C tests, and LDL-C level at the first test, are presented as the mean and standard deviation (SD). Multivariate logistic regression analysis was performed to analyze the factors affecting drug switching, resulting in adjusted odds ratios for each selected independent variable with a 95% confidence interval (CI).

To analyze the differences in clinical effectiveness, the mean LDL-C levels at the first and second examinations and the mean differences between the two examinations are presented as means with 95% CI for each patient group. The DID method is a commonly employed methodology in which outcomes before and after an event are compared between a study group that has experienced the event and a control group that has not, allowing the researcher to control for background changes in outcomes [23]. Therefore, this study performed a DID analysis to compare changes in LDL-C values depending on whether a drug switch (event) had occurred. To determine the effect of drug switching on LDL-C changes between the first and second examinations, the interaction term of examination time (dichotomous variable) × switch status (dichotomous variable) was included in the model to assess the significance of the DID regression results between the two groups. The unadjusted results (crude) and adjusted DID results were calculated, controlling for time-varying variables. Using Tukey’s boxplot, LDL-C outliers in switchers and non-switchers were defined as values outside the range (Q1–1.5 IQR, Q3 + 1.5 IQR) [24]. A two-sided *p*-value of < 0.05 was considered significant. All the statistical analyses were performed using SAS version 9.4 (SAS Institute, Cary, NC, USA).

### Ethical statement

This study was reviewed and approved by the Institutional Review Board of Sungkyunkwan University (IRB No. SKKU2021-05–010). The study analyzed publicly available data; hence, participant consent was not obtained and all personal information was de-identified by the NHIS prior to public release.

## Results

**Fig. 1:**
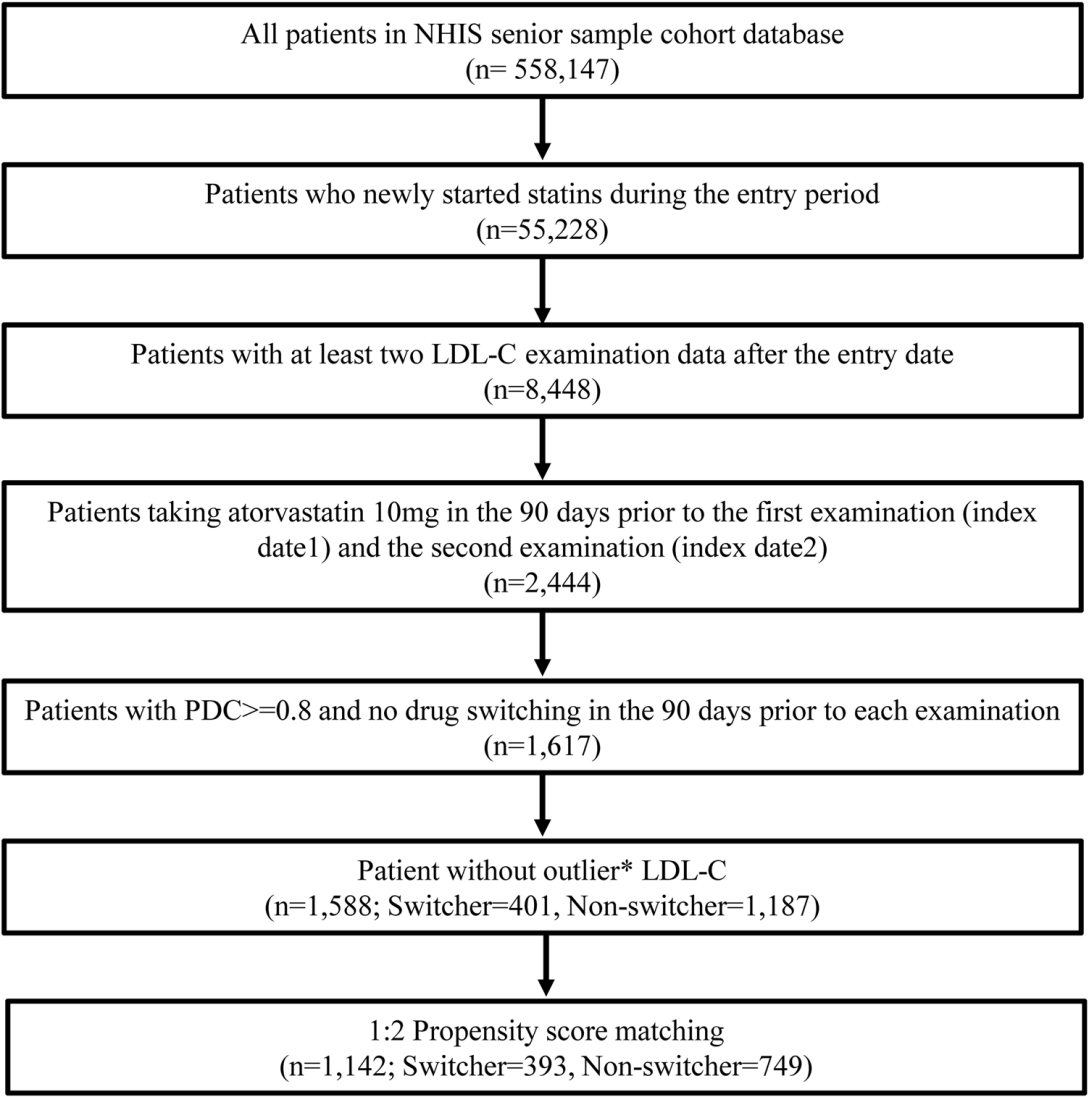
Study population flowchart. NHIS, National Health Insurance Service; LDL-C, low-density lipoprotein-cholesterol *Outlier was defined as values outside the range (Q1–1.5 IQR, Q3 + 1.5 IQR)

Of the 558,147 patients in the NHIS-senior cohort, 1,588 were newly initiated on atorvastatin 10 mg, comprising 1,187 non-switchers and 401 switchers (Fig. 1). In both groups, more than 50% of the patients were between 70 and 74 years of age, with mean ages of 74.3 and 74.5 years for non-switchers and switchers, respectively (standard mean difference [SMD] = 0.061). Unlike non-switchers, switchers were more likely to be women (57.0% *vs*. 62.1%, SMD = -0.103), and non-switchers were more likely to be in the upper income quintile (57.0% *vs*. 51.9%, SMD = 0.102). The time between the first and second examinations was 725.6 days (SD = 182.3 days) for non-switchers and 764.2 days (SD = 189.9 days) for switchers, with switchers experiencing a longer time interval than non-switchers (SMD = -0.207). After 1:2 propensity score matching, most characteristics were found to be similar between the two groups. However, the switcher group showed a slightly longer interval between examinations than that of the non-switcher group (739.1 *vs*. 759.7 days, SMD = -0.110), along with a slightly higher prevalence of hypertension (62.2% vs. 69.2%, SMD = -0.148) (Table 1).

**Table 1:** Baseline characteristics

Variables	Overall cohort					After matching				
	**Non-switcher (*n* = 1187)**		**Switcher (*n* = 401)**		**SMD**	**Non-switcher (*n* = 749)**		**Switcher (*n* = 393)**		**SMD**
	**No. of patients**	**%**	**No. of patients**	**%**		**No. of patients**	**%**	**No. of patients**	**%**	
**Age groups, Mean (SD)**	74.3	3.95	74.5	4.0	-0.061	74.3	3.8	74.6	4.0	-0.057
65–69	31	2.6	12	3.0	-0.023	14	1.9	8	2.0	-0.012
70–74	724	61.0	234	58.4	0.054	461	61.6	234	59.5	0.041
75–80	299	25.2	95	23.7	0.035	182	24.3	93	23.7	0.015
80–84	108	9.1	53	13.2	-0.131	82	11.0	51	13.0	-0.062
≥ 85	25	2.1	7	1.8	0.026	10	1.3	7	1.8	-0.036
**Sex**										
Female	677	57.0	258	62.1	-0.103	459	61.3	243	61.8	-0.011
**Household income**										
Low	217	18.3	86	21.2	-0.073	155	20.7	82	20.9	-0.004
Middle	294	24.8	110	26.9	-0.049	213	28.4	107	27.2	0.027
High	676	57.0	220	51.9	0.102	381	50.9	204	51.9	-0.021
**Charlson comorbidity index score**										
0	442	37.2	153	36.7	0.012	277	37.0	144	36.6	0.007
1	398	33.5	142	34.4	-0.019	258	34.5	135	34.4	0.002
2	212	17.9	75	18.0	-0.003	127	17.0	71	18.1	-0.029
≥ 3	135	11.4	46	11.0	0.013	87	11.6	43	10.9	0.021
**Obesity**	462	38.9	163	40.7	-0.035	307	41.0	159	40.5	0.011
**Lab test gap (day), Mean (SD)**	725.6	182.3	764.2	189.9	-0.207	739.1	181.1	759.7	193.3	-0.110
**1st LDL-C (mg/dL), Mean (SD)**	80.3	20.7	81.0	21.8	-0.034	80.9	21.1	80.9	20.6	0.003
**Co-medication**										
Cardiovascular & endocrine	162	13.7	52	13.0	0.020	100	13.4	50	12.7	0.019
Steroid & hormones	323	27.2	118	29.4	-0.049	202	27.0	118	30.0	-0.068
**Comorbidity**										
Ischemic cardiovascular disease	165	13.9	40	10.0	0.121	65	8.7	38	9.7	-0.034
Stroke	120	10.1	27	6.7	0.122	48	6.4	25	6.4	0.002
Arteriosclerosis	6	0.5	1	0.3	0.042	1	0.1	1	0.3	-0.028
Hypertension	719	60.6	276	68.8	-0.173	466	62.2	272	69.2	-0.148
Diabetes mellitus	308	26.0	102	25.4	0.012	207	27.6	100	25.4	0.050
Congestive heart failure	10	0.8	2	0.5	0.044	7	0.9	2	0.5	0.050

*SMD* Standardized mean difference, *SD* Standard deviation, *LDL-C* Low-density lipoprotein-cholesterol

Logistic regression analysis was performed to identify the factors that could affect drug switching. Age, sex, income level, and CCI score did not significantly affect drug switching. However, the odds of switching were significantly lower with an original atorvastatin drug formulation taken 90 days before the first visit than that with a generic formulation, with an odds ratio of 0.31 (95% CI [0.21, 0.46]). However, the odds of switching were 6.83 times higher (95% CI [4.66, 10.02]) if a different medical institution prescribed atorvastatin 10 mg 90 days before the first and second visits (Table 2).

**Table 2:** Multivariable logistic regression analysis of factors for switching

Variables	Adjusted odds ratio	(95% CI)
**Age**		
< 65	1.02	(0.37–2.79)
70–74	reference	
75–80	0.92	(0.67–1.27)
80–84	1.10	(0.73–1.67)
≥ 85	1.39	(0.48–4.05)
**Sex**		
Female	0.93	(0.71–1.23)
**Income**		
Low	reference	
Middle	0.97	(0.66–1.43)
High	1.05	(0.74–1.48)
**Obesity**		
Yes	0.96	(0.73–1.26)
**Charlson comorbidity index score**		
0	reference	
1	1.17	(0.84–1.64)
2	1.36	(0.89–2.08)
≥ 3	1.47	(0.85–2.57)
**1st period medication**		
Generic	reference	
Original	0.31	(0.20–0.48)
**Switching medical institution**		
Yes	6.83	(4.66–10.02)
**1st period medical institution**		
Hospital	reference	
Clinic	0.91	(0.65–1.29)
**Co-medication**		
Cardiovascular & endocrine	0.86	(0.58–1.29)
Steroid & hormones	1.12	(0.84–1.51)
**Comorbidity**		
Hypertension	1.09	(0.80–1.49)
Diabetes mellitus	0.69	(0.47–1.01)

*CI* Confidence interval

The mean changes in LDL-C values between examinations were 0.73 mg/dL (95% CI [-0.86, 2.32]) and 0.46 mg/dL (95% CI [-1.73, 2.65]) in the non-switcher and switcher groups, respectively, with no statistically significant change in either group. A DID of the mean change between the non-switcher and switcher groups showed no significant difference in LDL-C change, with an adjusted DID of 0.42 mg/dL (95% CI [-2.29, 3.13]), adjusted for concomitant medications and comorbidities (Table 3).

**Table 3:** Comparison of differences in low-density lipoprotein-cholesterol levels

	**Non-switcher (*n* = 749)**		**Switcher (*n* = 393)**	
**Outcomes**	**Mean**	**(95% CI)**	**Mean**	**(95% CI)**
1st LDL-C, mg/dL	80.92	(79.32, 82.52)	80.86	(78.64, 83.07)
2nd LDL-C, mg/dL	81.65	(80.05, 83.25)	81.32	(79.11, 83.53)
Mean difference, mg/dL	0.73	(-0.86, 2.32)	0.46	(-1.73, 2.65)
Crude DID, mg/dL	0.27		(-2.44, 2.97)	
Adjusted DID^a^, mg/dL	0.42		(-2.29, 3.13)	

*CI* Confidence interval, *LDL-C* Low-density lipoprotein-cholesterol, *DID* Difference-in-differences

^a^Adjusted for co-medication and comorbidities

In the non-switcher group, the original (brand-name) drug prescription was continued in 25.2% of patients (189 of 749) without a change, whereas generic drug prescriptions were continued in 74.8% of patients (560 of 749). Among the switchers, 12.7% (50 of 393) switched from original to generic drugs, 10.2% (40 of 393) switched from generic to original drugs, and 77.1% (303 of 393) switched from a generic to an alternate generic drug. Among the non-switchers, neither the original nor the generic groups showed a significant change in LDL-C levels. Among switchers, the original to generic (0.98 mg/dL; 95% CI [-3.15, 7.23]) and generic to another generic (1.07 mg/dL; [95% CI -1.34, 3.47]) subgroups exhibited minimal changes in LDL-C values (Table 4).

**Table 4:** Comparison of differences in low-density lipoprotein-cholesterol levels in subgroups of switch patterns

Outcomes	Mean value	(95% CI)
**Non-switcher (*n* = 749)**		
Original (*n* = 189)		
1st LDL-C, mg/dL	76.10	(74.47, 81.17)
v2nd LDL-C, mg/dL	76.26	(73.08, 79.78)
Mean difference, mg/dL	0.15	(-4.81, 2.03)
Generic (*n* = 560)		
1st LDL-C, mg/dL	83.56	(80.16, 83.78)
2nd LDL-C, mg/dL	82.82	(81.61, 85.22)
Mean difference, mg/dL	-0.74	(-0.42, 3.31)
**Switcher (*n* = 393)**		
Original → Generic (*n* = 50)		
1^st^ LDL-C, mg/dL	78.96	(82.44, 83.68)
2nd LDL-C, mg/dL	79.94	(74.48, 85.72)
Mean difference, mg/dL	0.98	(-3.15, 7.23)
Generic → Original (*n* = 40)		
1st LDL-C, mg/dL	83.56	(74.60, 88.35)
2nd LDL-C, mg/dL	76.00	(68.53, 82.27)
Mean difference, mg/dL	-7.56	(-12.34, 0.19)
Generic → Another generic (*n* = 303)		
1st LDL-C, mg/dL	83.56	(78.64, 83.83)
2nd LDL-C, mg/dL	84.56	(79.70, 84.90)
Mean difference, mg/dL	1.07	(-1.34, 3.47)

*CI* Confidence interval, *LDL-C* Low-density lipoprotein-cholesterol

Regarding hospital-level providers, 24.3% and 30.2% of patients switched medications when prescribed by the same hospital and the same clinic, respectively. Conversely, 80% and 68.2% of the patients switched from hospitals to clinics or from clinics to clinics, respectively. In the non-switcher group, LDL-C levels increased by 13.17 mg/dL (95% CI [-10.51, 36.85]) when switching from a hospital to a clinic and decreased by 8.82 mg/dL (95% CI [-8.56, 2.70]) when switching from a clinic to another clinic; however, these changes were not statistically significant. In the switcher group, a 5.22 mg/dL decrease in LDL-C level was observed in those who switched from a clinic to a hospital (95% CI [-16.35, 5.91]), although this change was not statistically significant (Table 5).

**Table 5:** Comparison of differences in low-density lipoprotein-cholesterol levels in the subgroup of medical institution utilization

**1st medical institution**		**2nd medical institution**	**Non-switcher (*n* = 749)**			**Switcher (*n* = 393)**		
			**No. of patients (%)**	**Mean difference, mg/dL**	**(95% CI)**	**No. of patients (%)**	**Mean difference, mg/dL**	**(95% CI)**
Hospital	→	Same Hospital	221 (75.68)	-0.44	(-3.46, 2.58)	71 (24.32)	1.14	(-3.52, 5.80)
Hospital	→	Other hospital	17 (54.84)	-8.82	(-19.62, 1.98)	14 (45.16)	-0.36	(-7.53, 6.81)
Hospital	→	Clinic	6 (20.00)	13.17	(-10.51, 36.85)	24 (80.00)	-1.96	(-8.60, 4.68)
Clinic	→	Same Clinic	476 (69.79)	1.74	(-0.32, 3.80)	206 (30.21)	0.93	(-1.93, 3.79)
Clinic	→	Other Clinic	28 (31.82)	-5.21	(-11.13, 0.71)	60 (68.18)	0.92	(-5.06, 6.90)
Clinic	→	Hospital	1 (5.26)	33.00	-	18 (94.74)	-5.22	(-16.35, 5.91)

*CI* Confidence interval

## Discussion

Herein, claims data were used to investigate the factors affecting drug switching and the differences in LDL-C levels between switchers and non-switchers among older patients who were newly prescribed atorvastatin. Within an observational period of approximately two years, 25.3% of patients switched to a different product with the same active ingredients. Drug switching was influenced by first taking the original drug and subsequently changing the prescribing medical institution. There was no significant difference in the LDL-C levels between the switcher and non-switcher groups. In addition, no statistically significant difference was observed in LDL-C changes between the switch scenarios and changing medical institutions. In the subgroup analysis of the changing medical institution scenario, the difference was considerable in certain instances; however, the small sample size hindered interpretation of the results.

Generics are approved because they are considered as effective as the original (brand-name) drug; therefore, these drugs can be used interchangeably. In real-world clinical practice, the perception of original and generic drugs has yet to converge [6, 7] despite frequent medication changes between brand names and generics or between generic and generic drugs [25]. In the present study, 25% of the patients (401 out of 1,588) switched within approximately two years. The most common switch scenario was from one generic drug to another (77.1%), which is similar to the findings of a previous study (80.3%) [5]. Nevertheless, there were no statistically significant changes in LDL-C values in the original-to-generic, generic-to-original, or generic-to-generic switching groups. Although there have been conflicting results on the clinical effectiveness of original and generic drugs, [10, 26, 27] our findings indicate that regardless of how the medications were switched, LDL-C levels were not significantly altered compared with those in patients who were taking the same medication. After adjusting for comorbidities and concomitant medications known to affect LDL-C control, drug switching had no significant effect on LDL-C levels. Other factors, such as diet, physical activity, and lifestyle changes, [28] may affect LDL-C changes; however, owing to the limitations of claims data, they could not be considered in this study. However, we controlled for confounders by matching and adjusting for the available variables; therefore, our results provide evidence of their interchangeability.

Other demographic factors did not significantly affect the incidence of drug switching in this study, although the initial use of brand-name medication was one of the strongest predictors of drug switching (odds ratio: 0.31; 95% CI [0.20, 0.48]). This finding is similar to those of several previous studies showing greater loyalty and trust in brand-name medications than generics [19, 20]. Furthermore, owing to the characteristics of the Korean healthcare system, which make it relatively easy to seek care from multiple medical institutions, [17] switching medical institutions was a strongly associated factor for drug switching. Other reasons for drug switching include negative perceptions of generics, political reasons, drug shortages, and patient desires, [7, 29–31] while neither comorbidity nor co-medication had a significant effect on drug switching.

Considering situations in which low-priced generic drugs continue to be released after the patent expires, several societal benefits allow healthcare providers to freely select various products if equivalent clinical effectiveness can be ensured between the original and generic drugs. Patients can reduce their drug costs by switching to a generic drug that is cheaper than the existing drugs, and pharmacies can prevent unnecessary resource wastage through flexible drug stockouts and efficient drug inventory management via substitution. The continued accumulation of clinical evidence based on real-world data, such as that in the present study, coupled with appropriate education and outreach to raise awareness among prescribers and patients, will afford multiple benefits, including reduced healthcare expenditure, efficient resource utilization, and improved access to healthcare.

### Strengths and limitations

Considering the strengths of this study, the NHIS-senior cohort data included drug product codes that facilitated the evaluation of drug changes. They linked health examination data, which allowed the measurement of LDL-C changes. Therefore, the clinical effects of several drug-switching scenarios were compared, making it difficult to conduct clinical trials. Moreover, to control for potential confounders, the study patients were matched using propensity score matching and the outcomes were evaluated using DID analysis. Nevertheless, the limitations of this study must be addressed. First, given that the present study was performed retrospectively using claims data, it was impossible to control for all the patients’ clinical characteristics at baseline, which could have led to selection bias. To control for this, we included patients newly introduced to statins and those with high adherence (MPR > 0.8), 90 days before each examination date to minimize selection bias. Second, patients who undergo regular medical examinations may have slightly different behavioral characteristics from those of the general population. Third, this study was limited by the inability to control for immeasurable confounders such as environmental and social policy factors, lifestyle changes and reasons for drug switching, which could not be measured using claims data [28, 32, 33]. Finally, during the study period, we focused solely on atorvastatin, a commonly used drug among the older population, and considered several generic formulations available in Korea. Therefore, caution must be exercised when generalizing the results of this study to all generic drugs.

## Conclusion

Collectively, our findings revealed that taking the original drug first decreased the odds of drug switching, and conversely, changing the medical institution increased the odds of switching. In addition, no significant differences in clinical effectiveness were observed across the various drug-switching scenarios or when comparing the original and generic drugs. Although these results pertain only to atorvastatin, they suggest that bioequivalent medications may be interchangeable in clinical practice. Future studies involving larger patient populations and multifaceted analyses are necessary to identify the potential differences.

### Supplementary Information

**Additional file 1: Supplementary Figure 1. **Study design.

## Data Availability

The data that support the findings of this study are available from the NHIS; however, restrictions apply to the availability of these data, which were used under license for the current study and are not publicly available. However, the data are available from the authors upon reasonable request and with permission from the NHIS.

## Abbreviations

CCI: Charlson comorbidity index
CI: Confidence interval
DID: Difference-in-differences
LDL-C: Low-density lipoprotein-cholesterol
NHIS: National Health Insurance Service
SD: Standard deviation
