# Supplementary material for: Factors influencing drug switching and changes in low-density lipoprotein-cholesterol levels with atorvastatin: a real-world observational study
Source: Lipids Health Dis. 2023 Sep 13;22:151. doi: 10.1186/s12944-023-01903-2 (PMC10498597; doi:10.1186/s12944-023-01903-2)
Supplement: Supplementary file 1 — Additional file 1: Supplementary Figure 1. Study design. [file 12944_2023_1903_MOESM1_ESM.docx]

**Supplementary Figure 1.** Study design

**
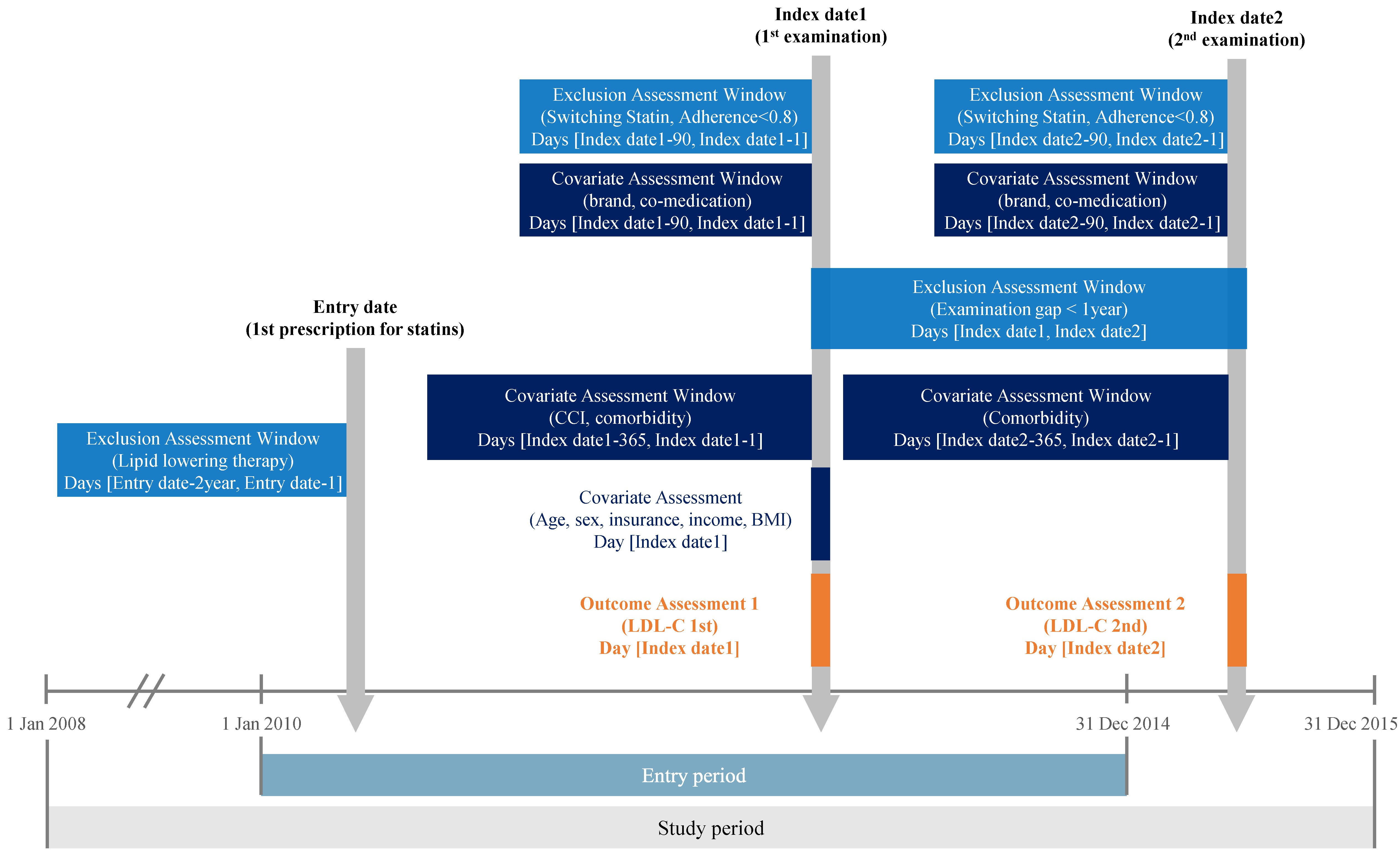
**

CCI, Charlson comorbidity index; LDL-C, low-density lipoprotein-cholesterol
